# Supplementary material for: Streamlined inactivation, amplification, and Cas13-based detection of SARS-CoV-2
Source: Nat Commun. 2020 Nov 20;11:5921. doi: 10.1038/s41467-020-19097-x (PMC7680145; doi:10.1038/s41467-020-19097-x)
Supplement: Supplementary file 1 — Supplementary Information [file 41467_2020_19097_MOESM1_ESM.pdf]

SUPPLEMENTARY INFORMATION

Streamlined inactivation, amplification, and Cas13-based detection of SARS-CoV-2

Arizti-Sanz & Freije *et al.*

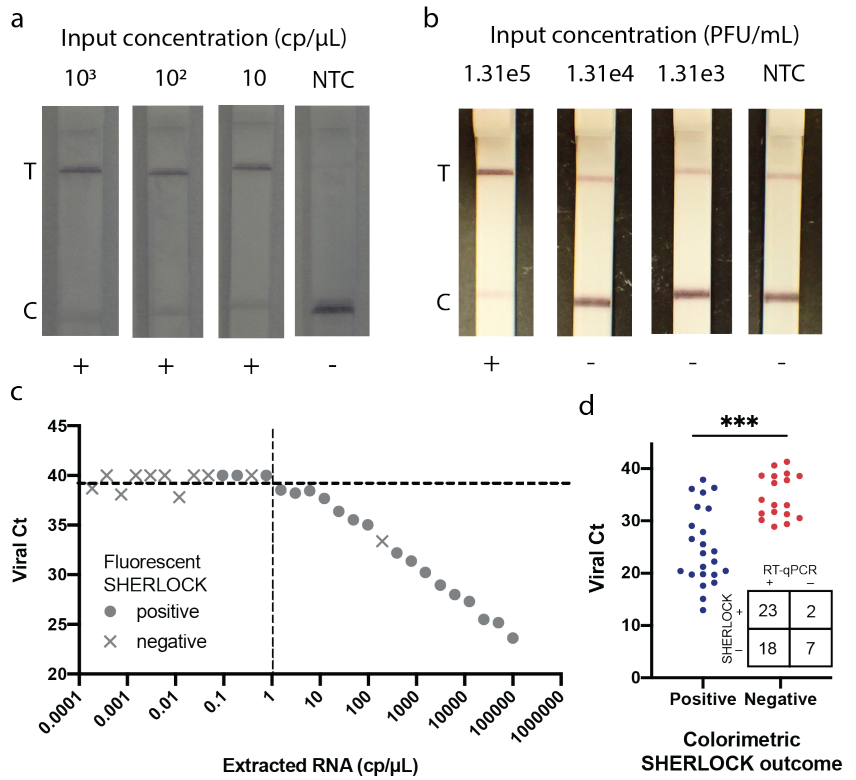

Supplementary Fig. 1. Additional two-step SHERLOCK testing.
a Colorimetric detection of synthetic DNA using two-step SHERLOCK after 3 h. NTC, non-template control; T, test line; C, control line. **b** Colorimetric detection of HUDSON-treated SARS-CoV-2 viral seedstock using two-step SHERLOCK after 3 h. NTC, non-template control; T, test line; C, control line. **c** Ct values of RT-qPCR for extracted RNA from SARS-CoV-2 seedstock at various concentrations. Symbol indicates the result of our two-step SHERLOCK assay performed side-by-side. The vertical line demarcates 1 cp/µL. The horizontal line demarcates samples with non-quantifiable Ct values (*i.e.,* no amplification), imputed as a Ct of 40. **d** Viral Ct values measured by RT-qPCR for extracted RNA from 41 RT-qPCR positive patient samples grouped by the result of the two-step SHERLOCK assay using a colorimetric, lateral flow-based readout. Inset is the concordance results of all samples tested by colorimetric two-step SHERLOCK and RT-qPCR. The association between viral Ct and two-step SHERLOCK outcome was assessed using a one-sided Wilcoxon rank sum test. ***, $p < 0.0001$. For **(c** and **d)** source data are provided as a Source Data file.

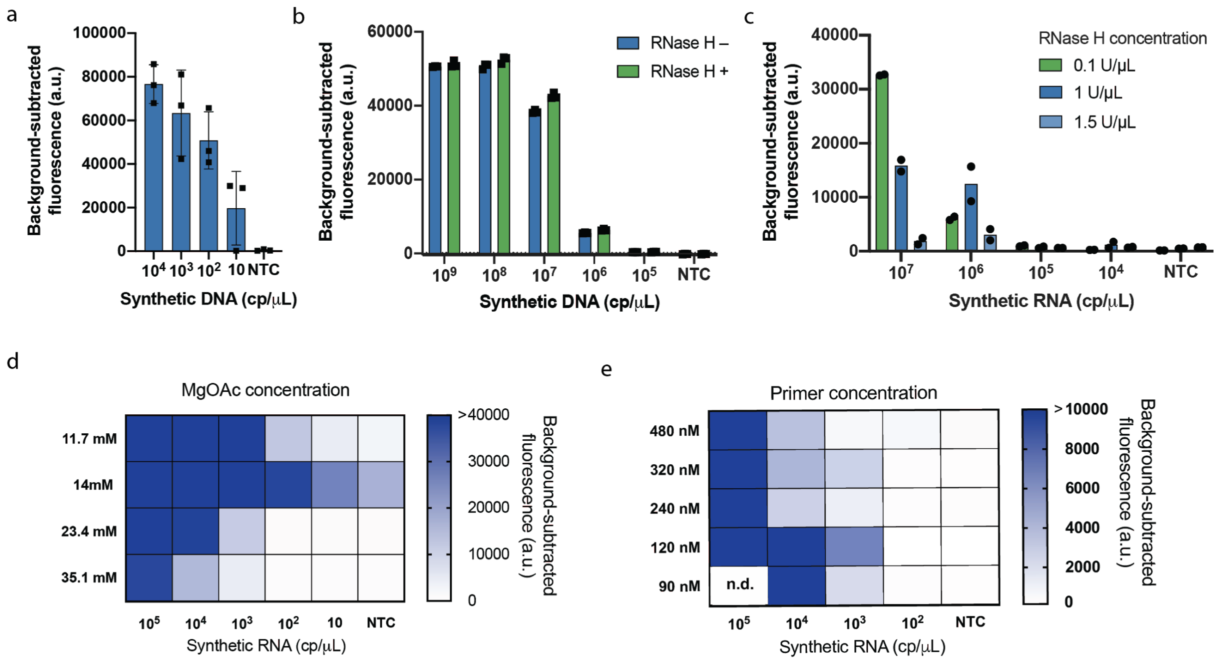

Supplementary Fig. 2. Optimization of single-step SHERLOCK for improved sensitivity.
a Background-subtracted fluorescence detected after the single-step SHERLOCK reaction was incubated for 3 h with DNA as input. **b** Background-subtracted fluorescence of the Cas13-detection reaction (no RPA enzymes) with 3 h incubation. RNase H+, final concentration of 0.1 U/μL. **c** Background-subtracted fluorescence of the Cas13-detection reaction (no RPA) after 3 h incubation with varying RNase H concentrations. **d** Background-subtracted fluorescence detected after the single-step reaction was incubated for 3 h with varying magnesium concentrations. **e** Background-subtracted fluorescence detected after the single-step reaction was incubated for 3 h with varying RPA primer concentrations. For **a-e**, NTC, non-template controls; error bars, s.d. for 2-3 technical replicates. All listed concentrations refer to concentration within the reaction mixture before addition of the oligonucleotide template. For (**a-c**), Center = mean and error bars = s.d. for 3 technical replicates. For **(a-e)** source data are provided as a Source Data file.

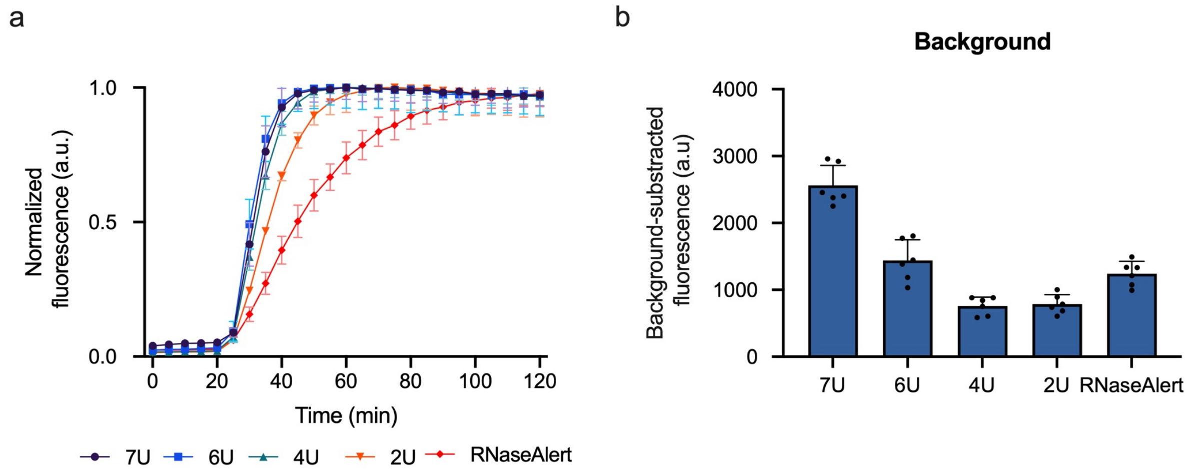

Supplementary Fig. 3. Optimization of fluorescent reporter.

a Single-step SHERLOCK normalized fluorescence (see Methods for details) over time using quenched poly-uracil FAM reporters of varying lengths or RNaseAlert with RNA input at 10^4 cp/µL. **b** Background-subtracted fluorescence of poly-uracil FAM reporters or RNaseAlert in single-step SHERLOCK after 3 h for non-template controls. For (**a** and **b**), Center = mean and error bars = s.d. for 3 (**a**) and 6 (**b**) technical replicates. Source data are provided as a Source Data file.

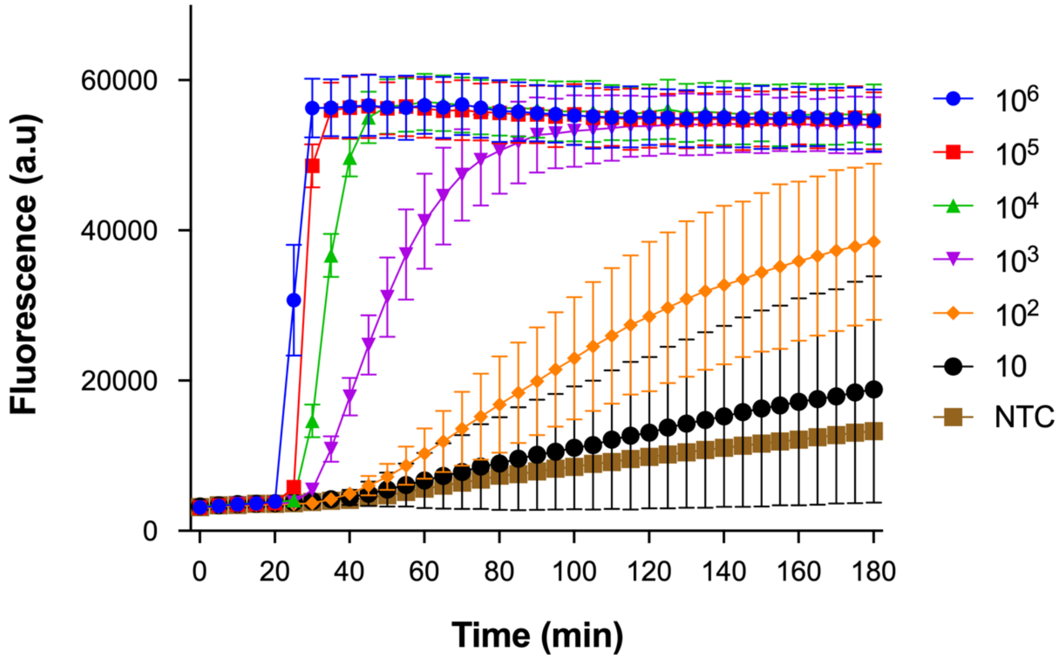

Supplementary Fig. 4. Single-step SHERLOCK time course.

Optimized single-step SHERLOCK assay fluorescence over time at varying RNA input concentrations. Background-subtracted fluorescence at 1 h is shown in Fig. 2e. NTC, non-template control; Center = mean and error bars = s.d. for 3 technical replicates. Note: error bars for NTC are present but small. Source data are provided as a Source Data file.

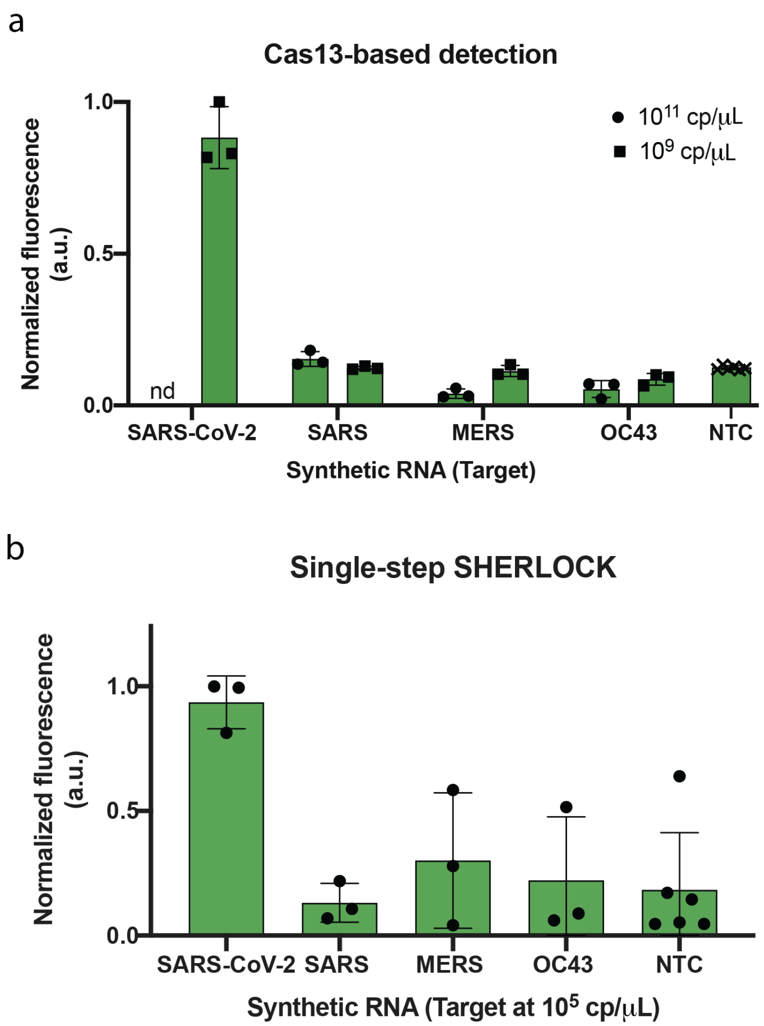

Supplementary Fig. 5. Single-step SHERLOCK specificity.

a Normalized fluorescence of Cas13-based detection using synthetic RNA targets of four human-infecting coronaviruses as the target input after 3 h. nd, not done. n = 3 replicates. **b** Normalized fluorescence of optimized single-step SHERLOCK using synthetic RNA targets of four human-infecting coronaviruses as target input after 3 h. NTC, non-template control; n = 3 replicates. error bars, s.d. for 3-6 technical replicates. For (**a** and **b**), Center = mean and error bars = s.d. for 3 technical replicates. Source data are provided as a Source Data file.

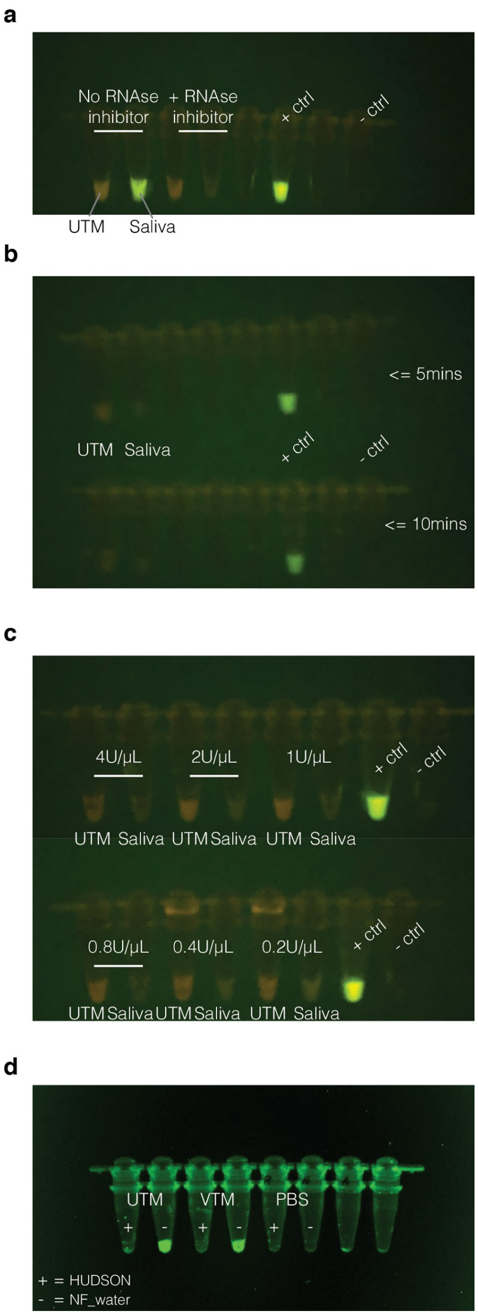

Supplementary Fig. 6. HUDSON optimization experiments.
a Samples were treated with 100 mM TCEP and 1 mM EDTA and subjected to a 20 min heating step at 50 ˚C. RNase inhibitor, 4 U/μL, unless otherwise specified. **b** Samples were treated with 100 mM TCEP, 1mM EDTA, and 4 U/μL RNase inhibitor. **c** Samples were treated with 100 mM TCEP and 1 mM EDTA and subjected to a 5 min heating step at 50 ˚C. **d** Samples in UTM, VTM, or PBS were treated with 100 mM TCEP and 1 mM EDTA or nuclease-free water and subjected to a 5 min heating step at 50 ˚C. For **a-d**, positive and negative controls undergo no treatment. RNaseAlert (final concentration: 200 nM) was added immediately after the heating step and is used to measure the RNase activity.

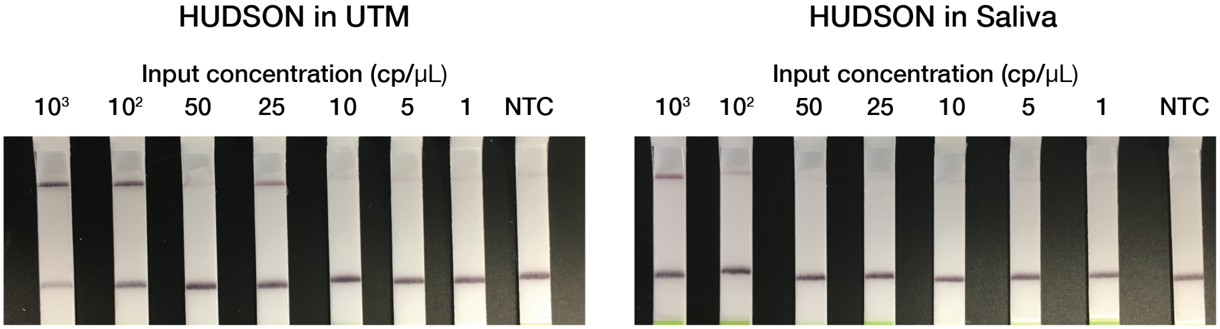

Supplementary Fig. 7. SHINE for UTM and saliva with colorimetric detection.
SHINE with colorimetric readout using synthetic RNA template spiked into UTM (left) and saliva (right) after the initial HUDSON heating step.

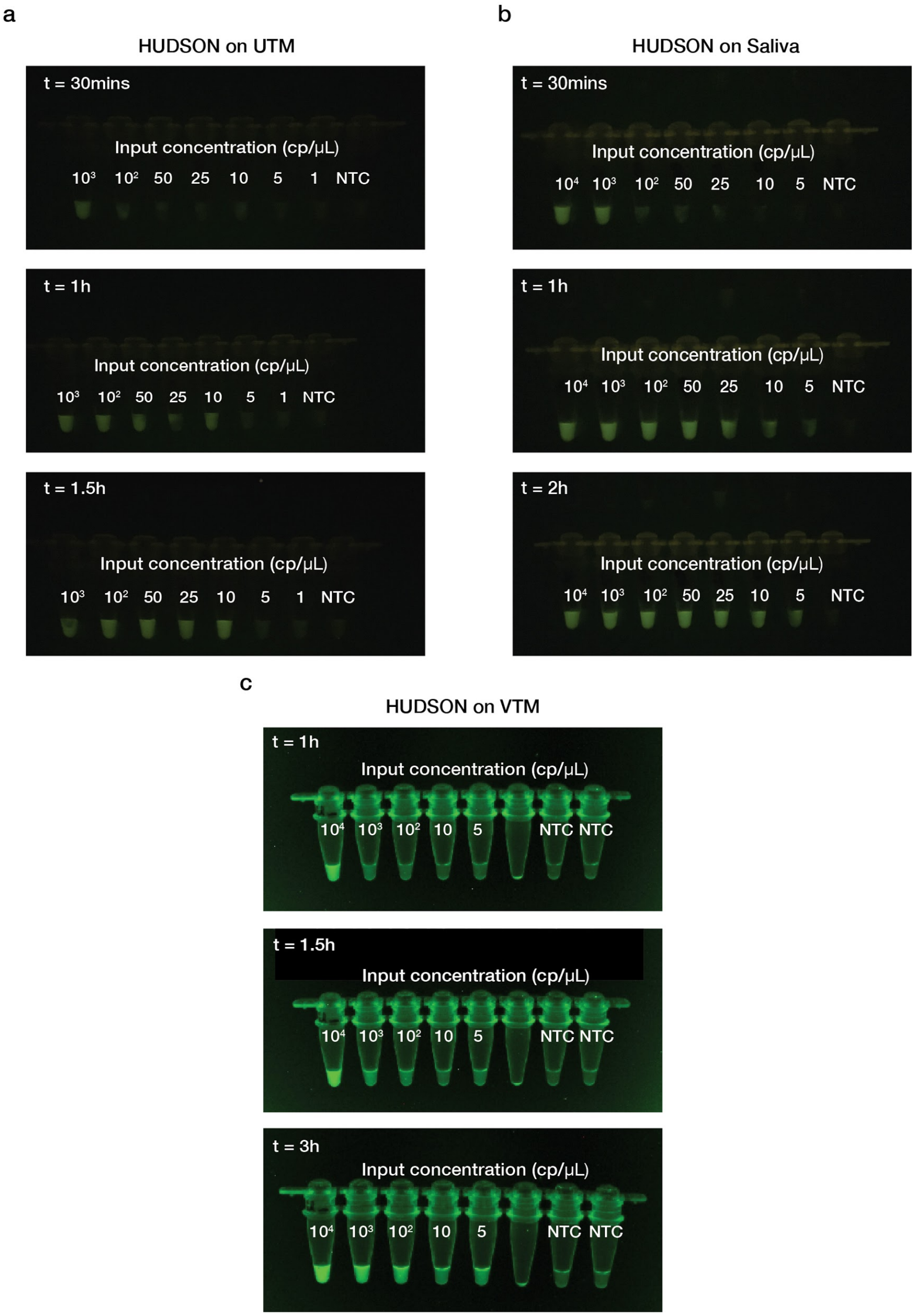

Supplementary Fig. 8. SHINE for UTM, VTM, and saliva with in-tube fluorescent detection. **a-c** SHINE with in-tube readout using synthetic RNA template spiked into UTM (**a**), saliva (**b**) and VTM (**c**) after the initial HUDSON heating step. Transilluminator or GelDoc images were captured using a smartphone camera. NTC, non-template control.

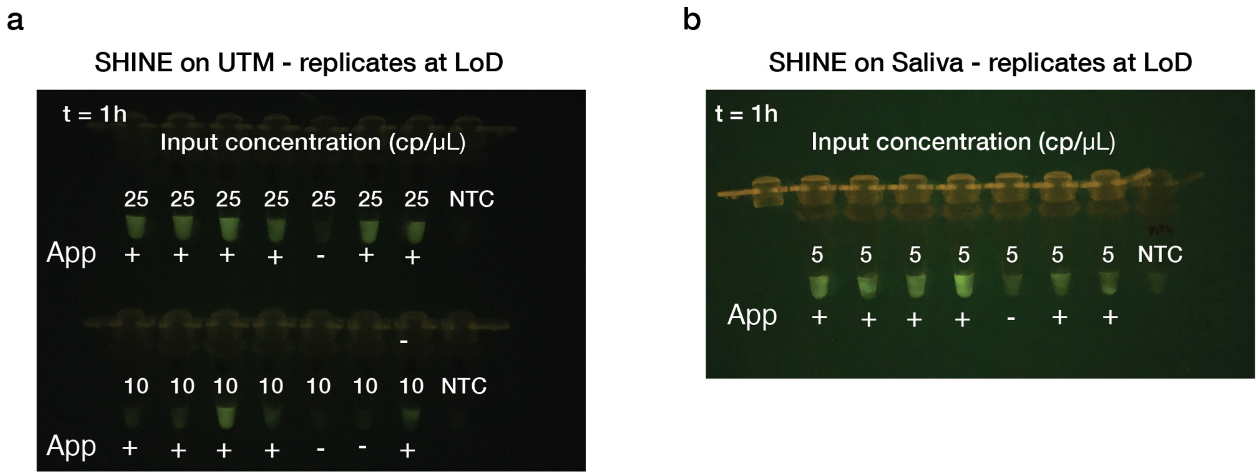

Supplementary Fig. 9. Limits of detection of SHINE on UTM and saliva.
a-b SHINE with in-tube readout using synthetic RNA template spiked into UTM (**a**) and saliva (**b**) after the initial HUDSON heating step. Transilluminator images were captured using a smartphone camera and analyzed by the companion smartphone application (App).

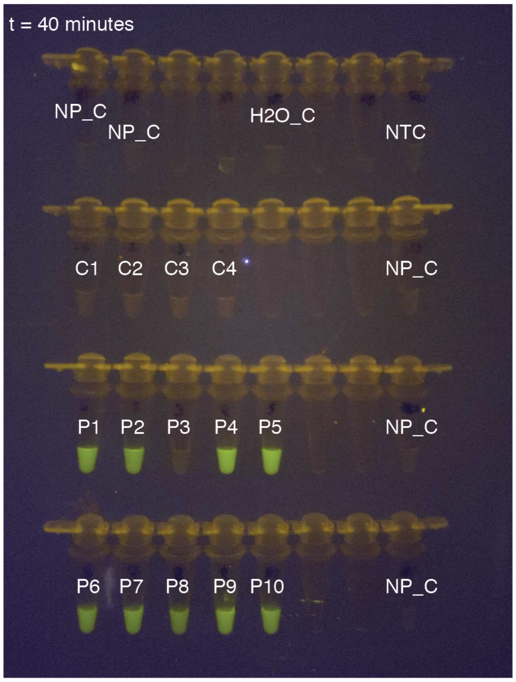

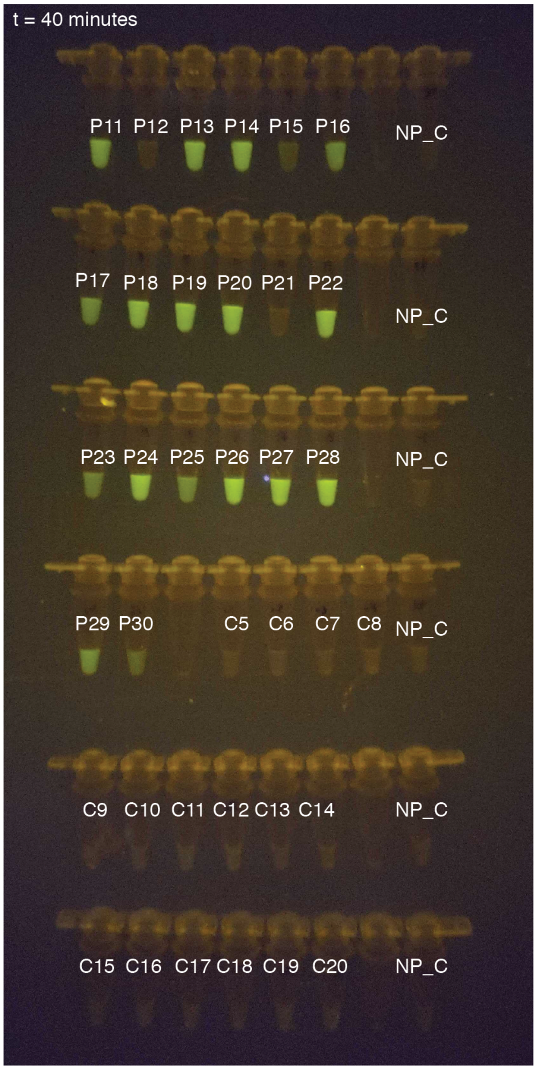

NP_C = HUDSON-treated healthy nasal fluid
H2O_C = HUDSON-treated nuclease-free water
NTC = Nuclease-free water

Supplementary Fig. 10. SHINE on unextracted patient samples.
NP swabs in UTM were used as input into the SHINE assay. Transilluminator images were captured using a smartphone camera after 40 min of single-step SHERLOCK incubation.

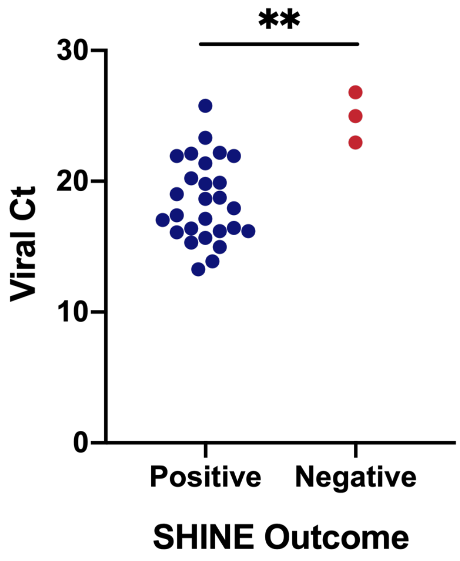

Supplementary Fig. 11. SHINE's ability to detect viral RNA is significantly associated with the RT-qPCR threshold cycle.
Viral Ct values measured by SARS-CoV-2 RT-qPCR of extracted RNA from 30 patient NP samples grouped by the result of the SHINE assay. The association between viral Ct and SHINE outcome was assessed using a one-sided Wilcoxon rank sum test. **, p = 0.0017. Source data are provided as a Source Data file.

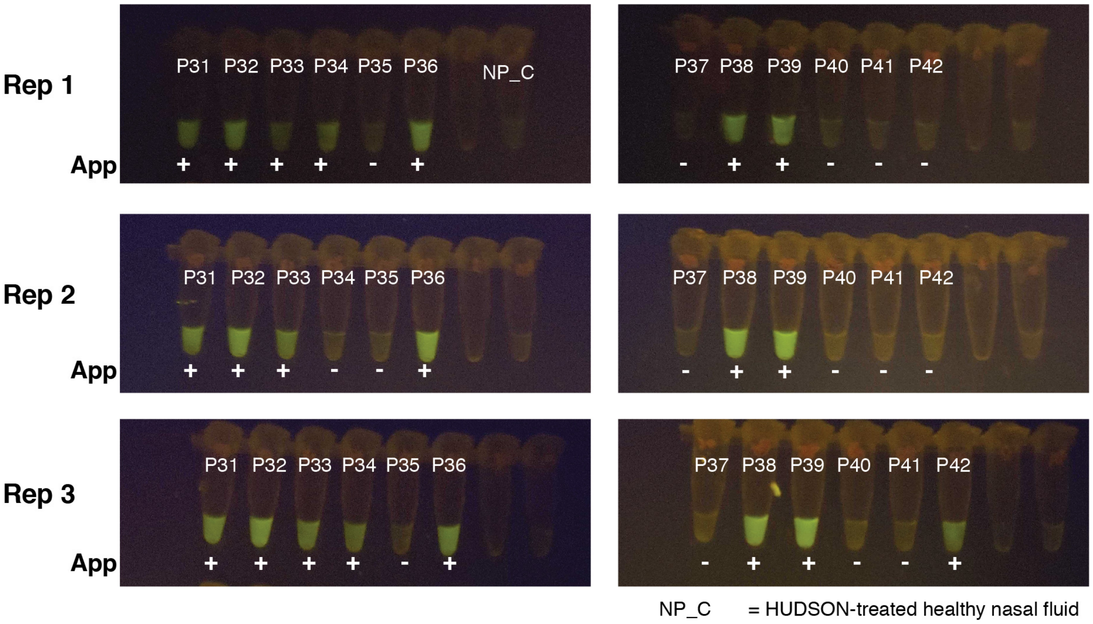

Supplementary Fig. 12. SHINE on unextracted patient samples.
NP swabs in VTM were used as input into the SHINE assay. Transilluminator images were captured using a smartphone camera after 40 min of single-step SHERLOCK incubation. Samples were tested in triplicate. Rep, replicate. App, readout with smartphone application.

Sample name	Sample ID	Sample type	Viral Ct	Viral quantity (cp/µL)	Figures
E1	ON507	S & NP in VTM	12.94	-	S1d
E2	KW326	S & NP in VTM	19.81	-	S1d
E3	KW298	S & NP in VTM	17.63	-	S1d
E4	KW151	NP in VTM	29.06	-	S1d
E5	EK220	NP in VTM	19.75	-	S1d
E6	OS555	S & NP in VTM	19.70	-	S1d
E7	EK272	NP in VTM	31.43	-	S1d
E8	EK271	NP in VTM	33.01	-	S1d
E9	EK239	NP in VTM	38.67	-	S1d
E10	EK219	NP in VTM	37.77	-	S1d
E11	EK204	NP in VTM	37.87	-	S1d
E12	KW311	S & NP in VTM	30.18	-	S1d
E13	OY213	NP in VTM	31.73	-	S1d
E14	OS533	S & NP in VTM	36.33	-	S1d
E15	ON444	S & NP in VTM	38.56	-	S1d
E16	ON356	S & NP in VTM	32.36	-	S1d
E17	ON1010	S & NP in VTM	15.11	-	S1d
E18	ON969	S & NP in VTM	18.19	-	S1d
E19	ON752	S & NP in VTM	20.46	-	S1d

E20	ON964	S & NP in VTM	20.43	-	S1d
E21	ON997	NP in VTM	21.21	-	S1d
E22	ON802	S & NP in VTM	22.23	-	S1d
E23	ON915	NP in VTM	23.81	-	S1d
E24	ON829	S & NP in VTM	24.17	-	S1d
E25	ON718	S & NP in VTM	25.52	-	S1d
E26	ON686	S & NP in VTM	26.55	-	S1d
E27	ON848	S & NP in VTM	27.89	-	S1d
E28	ON1012	S & NP in VTM	28.91	-	S1d
E29	ON1014	S & NP in VTM	29.4	-	S1d
E30	ON900	S & NP in VTM	30.55	-	S1d
E31	ON700	S & NP in VTM	31.27	-	S1d
E32	ON973	NP in VTM	32.71	-	S1d
E33	OS597	S & NP in VTM	33.05	-	S1d
E34	OS634	S & NP in VTM	34.03	-	S1d
E35	OY358	NP in VTM	35.42	-	S1d
E36	OY396	NP in VTM	36.12	-	S1d
E37	OY351	NP in VTM	37.25	-	S1d
E38	OY349	NP in VTM	38.58	-	S1d
E39	OY975	S & NP in VTM	39.05	-	S1d

E40	ON646	NP in VTM	40.6	-	S1d
E41	ON986	NP in VTM	41.33	-	S1d
E42	MA_MGH_00155	NP in UTM	25.989	892.22	2g, h
E43	MA_MGH_00156	NP in UTM	17.753	85,470.64	2g, h
E44	MA_MGH_00157	NP in UTM	17.235	113,800.532	2g, h
E45	MA_MGH_00159	NP in UTM	20.861	15,298.96	2g, h
E46	MA_MGH_00160	NP in UTM	20.006	24,515.988	2g, h
E47	MA_MGH_00166	NP in UTM	25.548	1,136.696	2g, h
P1	MA_MGH_00441	NP in UTM	22.109	52787.064	3g, h
P2	MA_MGH_00442	NP in UTM	25.764	4386.186	3g, h
P3	MA_MGH_00443	NP in UTM	26.809	2154.246	3g, h
P4	MA_MGH_00445	NP in UTM	16.429	3390658.2	3g, h
P5	MA_MGH_00446	NP in UTM	17.136	2022960.6	3g, h
P6	MA_MGH_00447	NP in UTM	16.39	3426433.56	3g, h
P7	MA_MGH_00453	NP in UTM	21.935	68312.187	3g, h
P8	MA_MGH_00454	NP in UTM	21.936	68293.593	3g, h
P9	MA_MGH_00456	NP in UTM	15.678	5660253.54	3g, h
P10	MA_MGH_00458	NP in UTM	23.331	25530.948	3g, h
P11	MA_MGH_00459	NP in UTM	15.313	7329902.04	3g, h
P12	MA_MGH_00460	NP in UTM	24.979	7977.141	3g, h
P13	MA_MGH_00461	NP in UTM	16.2	3914305.92	3g, h
P14	MA_MGH_00463	NP in UTM	17.05	2157841.26	3g, h
P15	MA_MGH_00464	NP in UTM	20.234	227657.529	3g, h
P16	MA_MGH_00465	NP in UTM	16.102	4201061.4	3g, h
P17	MA_MGH_00466	NP in UTM	17.933	1151605.125	3g, h
P18	MA_MGH_00467	NP in UTM	19.023	541607.094	3g, h
P19	MA_MGH_00468	NP in UTM	18.661	689269.86	3g, h

P20	MA_MGH_00469	NP in UTM	14.983	9240984	3g, h
P21	MA_MGH_00471	NP in UTM	22.963	33130.494	3g, h
P22	MA_MGH_00472	NP in UTM	13.271	30975183	3g, h
P23	MA_MGH_00473	NP in UTM	18.757	645122.16	3f, g, h
P24	MA_MGH_00474	NP in UTM	17.397	1682047.8	3f, g, h
P25	MA_MGH_00475	NP in UTM	19.896	289419.75	3f, g, h
P26	MA_MGH_00476	NP in UTM	16.181	3970140.3	3f, g, h
P27	MA_MGH_00477	NP in UTM	19.812	305659.512	3f, g, h
P28	MA_MGH_00479	NP in UTM	13.868	20324826	3f, g, h
P29	MA_MGH_00480	NP in UTM	21.362	102417.507	3g, h
P30	MA_MGH_00481	NP in UTM	22.187	57196.188	3g, h
P31	BocaBio_58	NP in VTM	26.778	378.502	S12
P32	BocaBio_59	NP in VTM	23.752	3107.53	S12
P33	BocaBio_61	NP in VTM	27.356	253.299	S12
P34	BocaBio_66	NP in VTM	25.549	605.047	S12
P35	BocaBio_67	NP in VTM	30.27	20.938	S12
P36	BocaBio_69	NP in VTM	22.577	5073.833	S12
P37	BocaBio_70	NP in VTM	31.299	9.99	S12
P38	BocaBio_73	NP in VTM	19.235	55318.152	S12
P39	BocaBio_78	NP in VTM	16.798	316178.75	S12
P40	BocaBio_80	NP in VTM	32.006	6.924	S12
P41	BocaBio_82	NP in VTM	28.433	77.379	S12
P42	BocaBio_83	NP in VTM	26.303	353.353	S12

Supplementary Table 1: Patient sample information.
For samples E42-E47 and P1-P30, the viral quantity listed below is adjusted as sample was diluted 1:3 for input into in-house RT-qPCR assay. For sample type “S & NP in VTM”, saliva and nasal specimens were combined during nucleic acid extraction. NP, nasopharyngeal swab; S, saliva; UTM, universal viral transport medium; VTM, viral transport medium.

Sample name	Sample ID	Sample type	Viral Ct	Viral quantity (cp/µL)	Figures
C1	EK276	NP in VTM	-	-	S1d
C2	OY314	NP in VTM	-	-	S1d
C3	OS572	S & NP in VTM	-	-	S1d
C4	ON513	S & NP in VTM	-	-	S1d
C5	ON1179	S & NP in VTM	-	-	S1d
C6	ON1180	S & NP in VTM	-	-	S1d
C7	ON1181	S & NP in VTM	-	-	S1d
C8	ON1182	S & NP in VTM	-	-	S1d
C9	ON1183	S & NP in VTM	-	-	S1d
C10	MA_MGH_004 11	NP in UTM	-	-	3g, h
C11	MA_MGH_004 12	NP in UTM	-	-	3g, h
C12	MA_MGH_004 13	NP in UTM	-	-	3g, h
C13	MA_MGH_004 14	NP in UTM	-	-	3g, h
C14	MA_MGH_003 20	NP in UTM	-	-	3f, g, h
C15	MA_MGH_003 21	NP in UTM	-	-	3f, g, h
C16	MA_MGH_003 22	NP in UTM	-	-	3f, g, h
C17	MA_MGH_003 23	NP in UTM	-	-	3f, g, h

C18	MA_MGH_003 24	NP in UTM	-	-	3g, h
C19	MA_MGH_003 25	NP in UTM	-	-	3g, h
C20	MA_MGH_003 26	NP in UTM	-	-	3g, h
C21	MA_MGH_003 27	NP in UTM	-	-	3g, h
C22	MA_MGH_003 28	NP in UTM	-	-	3g, h
C23	MA_MGH_003 29	NP in UTM	-	-	3g, h
C24	MA_MGH_003 38	NP in UTM	-	-	3g, h
C25	MA_MGH_003 53	NP in UTM	-	-	3g, h
C26	MA_MGH_003 54	NP in UTM	-	-	3g, h
C27	MA_MGH_003 55	NP in UTM	-	-	3g, h
C28	MA_MGH_003 56	NP in UTM	-	-	3g, h
C29	MA_MGH_003 57	NP in UTM	-	-	3g, h

Supplementary Table 2: COVID-19 negative patient sample information.
C1-C9 samples were collected from symptomatic, suspected COVID-19-positive patients but tested negative for SARS-CoV-2 RNA by RT-qPCR. C10-C29 samples were collected prior to the start of the coronavirus pandemic, and therefore are assumed not to contain SARS-CoV-2 RNA. NP, nasopharyngeal swab; S, saliva; UTM, universal viral transport medium.

Reagent	Reaction	Source	Stock Concentration	Notes
EDTA	HUDSON	Thermo Fisher Scientific™	0.5 M	
TCEP-HCl	HUDSON	Thermo Fisher Scientific™	0.5 M	
Universal Viral Transport Medium (UTM)	HUDSON	BD	N/A	
Saliva, Pooled Human Donors	HUDSON	Lee Biosolutions, Inc.	N/A	
RNase Inhibitor	HUDSON; RPA; SHERLOCK	NEB®	40 U/μL	Murine
HiScribe™ T7 High Yield RNA Synthesis Kit	IVT	NEB®	N/A	
RNAClean XP	IVT	Beckman Coulter, Inc.	N/A	
RNase-Free DNase I	IVT	QIAGEN		
T7 Promoter ssDNA Primer	IVT	Integrated DNA Technologies™		Supplementary Table 4 for sequences
Ambion® Linear Acrylamide	PCR	Thermo Fisher Scientific™	5 mg/mL	
AMPure XP	PCR	Beckman Coulter, Inc.	N/A	
Inter-amplicon Primer	PCR	Integrated DNA Technologies™	5 μM	Supplementary Table 4 for sequences
MagMAX™ *mir*Vana™ Total RNA Isolation Kit	PCR	Thermo Fisher Scientific™	N/A	
PCR Primers (forward, reverse)	PCR	Integrated DNA Technologies™		Supplementary Table 4 for sequences
Power SYBR® Green Master Mix	PCR	Thermo Fisher Scientific™	N/A	
TaqPath™ 1-Step RT-qPCR Master Mix	PCR	Thermo Fisher Scientific™	4×	

TURBO™ DNase	PCR	Thermo Fisher Scientific™	2 U/µL	
Magnesium Acetate (MgOAc)	RPA	TwistDx™	280 mM	TwistAmp® Basic Kit
RevertAid Reverse Transcriptase	RPA	Thermo Fisher Scientific™	200 U/µL	
RNase H	RPA	NEB®	5 U/µL	
RPA Pellets (lyophilized)	RPA	TwistDx™	N/A	TwistAmp® Basic Kit
RPA Primers (forward, reverse)	RPA	Integrated DNA Technologies™	5 µM of each primer	Supplementary Table 4 for sequences
SuperScript IV Reverse Transcriptase	RPA	Thermo Fisher Scientific™	200 U/µL	Invitrogen
Synthetic DNA Target	RPA	Integrated DNA Technologies™	10^{10} copies/µL	Supplementary Table 4 for sequences
Synthetic RNA Target	RPA	N/A	10^{10} copies/µL	generated via IVT of synthetic DNA target
Nuclease-free Water	RPA; SHERLOCK	Thermo Fisher Scientific™	N/A	Invitrogen
Reaction Buffer (Optimized)	RPA; SHERLOCK	N/A	5×	0.1 M HEPES pH 8.0, 300 mM KCl, 25% PEG-8000
Reaction Buffer (Original)	RPA; SHERLOCK	N/A	5×	0.1 M HEPES pH 6.8, 300 mM NaCl, 25% PEG-8000, 25 uM DTT
Cas13a crRNA	SHERLOCK	Integrated DNA Technologies™	2 µM	Supplementary Table 4 for sequences
Cleavage Buffer (CB)	SHERLOCK	N/A	10×	400 mM Tris pH 7.5, 10 mM DTT
FAM Cleavage Reporter (Biotinylated)	SHERLOCK	Integrated DNA Technologies™	16 µM	Supplementary Table 4 for sequences
polyU FAM Cleavage Reporter (Quencher)	SHERLOCK	Integrated DNA Technologies™	2 µM	Supplementary Table 4 for sequences
*Lwa*Cas13a protein	SHERLOCK	GenScript®	0.5 mg/mL	Custom protein purification as described previously (23)
Magnesium Chloride (MgCl2)	SHERLOCK	Thermo Fisher Scientific™	1 M	Invitrogen

HybriDetect Assay Buffer	SHERLOCK	Milenia® Biotec	N/A	
HybriDetect 1 Lateral Flow Strips	SHERLOCK	Milenia® Biotec	N/A	
RNaseAlert® Substrate v2	SHERLOCK	Thermo Fisher Scientific™	2 µM	
rNTPs	SHERLOCK	NEB®	25 mM of each nucleotide	
Storage Buffer (SB)	SHERLOCK	N/A	1×	50 mM Tris pH 7.5, 600 mM NaCl, 5% glycerol, 2 mM DTT
T7 RNA Polymerase	SHERLOCK	Lucigen®	50 U/µL	NextGen®
Vero (CCL-81™)	VIRAL STOCK	ATCC®	N/A	Grivet kidney epithelial cells
Dulbecco's Modified Eagle Medium (DMEM), high glucose, pyruvate	VIRAL STOCK	Thermo Fisher Scientific™	N/A	Gibco®
Fetal Bovine Serum (FBS)	VIRAL STOCK	SAFC Biosciences, Inc.	N/A	
Vero C1008 (CRL-1586™)	VIRAL STOCK	ATCC®	N/A	Grivet kidney epithelial cells
Avicel® PH-101	VIRAL STOCK	MilliporeSigma	N/A	
Crystal Violet	VIRAL STOCK	MilliporeSigma	N/A	

Supplementary Table 3: Reagents used in either the optimized single-step assay or in the optimization process.

HUDSON = Heating Unextracted Diagnostic Samples to Obliterate Nucleases. IVT = *In-vitro* Transcription. PCR = Polymerase Chain Reaction. RPA = Recombinase Polymerase Amplification. SHERLOCK = Specific High Sensitivity Enzymatic Reporter Unlocking.

Reagent	Sequence	SARS-CoV-2 Gene Location
Cas13a crRNA	CUCUUCUUCAGGUUGAAGAGCAGCAGAA	Orf1ab
PCR Primer (forward)	GACCCCAAAATCAGCGAAAT	N1
PCR Primer (reverse)	TCTGGTTACTGCCAGTTGAATCTG	N1
PCR Probe	/FAM/ACCCCGCATTACGTTTGGTGGACC/BHQ1	
RPA Primer (forward)	CCAAGGTAAACCTTTGGAATTTGGTGCCAC	Orf1ab
RPA Primer (reverse)	ACTATCATCATCTAACCAATCTTCTTCTTG	Orf1ab
Synthetic MERS DNA Target	GCACCATGCTATTGCTTTAACGCTGAGGGTGATGCATC<br>TTGGTCTTCTACTATGATCTTCTCTCTTCACCCCGTCGA<br>GTGTGACGAGGAGTGTTCTGAAGTAGAGGCTTCAGATT<br>TAGAAGAAGGTGAATCAGAGTGCATTTCTGAGACTTCAA<br>CTGAACAAGTTGACGTTTCTCATGAGGTTTCTGACGACG<br>AGTGGGCTGCTGCAGTTGATGAAGCGTTCCCCCTCGAT<br>GAAGCAGAAGATGTTACTGAATCTGTGCAAGAAGAAGC<br>ACAACCAGTAGAAGTACCTGTTGAAGATATTGCGCAGG<br>TTGTCATAGCTGACACCTTACAGGAAACTCCTGTTGTGT<br>CTGATACTGTTGAAGTCCCACCGCAAGTGGTGAAACTT<br>CCGTCTGAACCTCAGACTATCCAGCCCGAGGTAAAAGA<br>AGTTGCACCTGTCTATGAGGCTGATACCGAACAGACAC<br>AGAGTGTTACTGTTA	
Synthetic OC43 DNA Target	ATATGGAGGAATTTTATGCTGTGGTGATTGATGCCATAG<br>AAGAGAAACTTTCTCCATGTAAGGAGCTTGAAGGTGTA<br>GGTGCTAAAGTTAGTGCCTTTTTACAGAAATTAGAGGAT<br>AATCCCCTATTTTTATTTGATGAGGCTGGCGAAGAAGTT<br>TTTGCTCCTAAATTGTATTGTGCCTTTACAGCTCCTGAA<br>GATGATGACTTTCTTGAGGAAAGTGATGTTGAAGAAGAT<br>GATGTAGAAGGTGAGGAAACTGATTTAACTATCACAAGT<br>GCTGGACAGCCTTGTGTTGCTAGTGAACAGGAGGAATC<br>TTCTGAAGTCTTAGAGGACACTTTGGATGATGGTCCAAG<br>TGTGGAGACATCTGATTCACAAGTTGAAGAAGATGTAGA<br>AATGTCGGATTTTGTTGATCTTGAATCTGTGATTCAGGA<br>TTATGAAAATGTTTGTTTTGAGTTTTATACTACAGAGCCA<br>GAATTTGT	

Synthetic SARS DNA Target	GAATGTGAGAATCACATTTGAGCTTGATGAACGTGTTGACAAAGTGCTTAATGAAAAGTGCTCTGTCTACACTGTTGAATCCGGTACCGAAGTTACTGAGTTTGCATGTGTTGTAGCAGAGGCTGTTGTGAAGACTTTACAACCAGTTTCTGATCTCCTTACCAACATGGGTATTGATCTTGATGAGTGGAGTGTAGCTACATTCTACTTATTTGATGATGCTGGTGAAGAAAACTTTTCATCACGTATGTATTGTTCCTTTTACCCTCCAGATGAGGAAGAAGAGGACGATGCAGAGTGTGAGGAAGAAGAAATTGATGAAACCTGTGAACATGAGTACGGTACAGAGGATGATTATCAAGGTCTCCCTCTGGAATTTGGTGCCTCAGCTGAAACAGTTCGAGTTGAGGAAGAAGAAGAGGAAGACTGGCTGGATGATACTACTGAGCAATCAGAGATTGAGCCAGAACCAGAA	
Synthetic SARS-CoV-2 DNA Target	GTGAGTTTAAATTGGCTTCACATATGTATTGTTCTTTCTACCCTCCAGATGAGGATGAAGAAGAAGGTGATTGTGAAGAAGAAGAGTTTGAGCCATCAACTCAATATGAGTATGGTACTGAAGATGATTACCAAGGTAAACCTTTGGAATTTGGTGCCACTTCTGCTGCTCTTCAACCTGAAGAAGAGCAAGAAGAAGATTGGTTAGATGATGATAGTCAACAAACTGTTGGTCAACAAGACGGCAGTGAGGACAATCAGACAACTACTATTCAAACAATTGTTGAGGTTCAACCTCAATTAGAGATGGAACTTACACCAGTTGTTCAGACTATTGAAGTGAATAGTTTTAGTGGTTATTTAAAACTTACTGACAATGTATACATTAAAAATGCAGACATTGTGGAAGAAGCTAAAAAGGTAAAACCAACAGTGGTTGTTAATGCAGCCAATGTTTACCTTAAACATGGAGGAGG	Orf1ab
T7 Promoter ssDNA Primer	GAAATTAATACGACTCACTATAGGG	dsDNA appended upstream of target
FAM Cleavage Reporter (Biotinylated)	/56-FAM/rUrUrUrUrUrUrUrUrUrUrUrUrU/3Bio/	
polyU (*i.e.*, 6U or 7U) FAM Cleavage Reporter (Quencher)	/56-FAM/rUrUrUrUrUrU(rU)/3IABkFQ/	

Supplementary Table 4: Oligonucleotides used in this study.
MERS = Middle East Respiratory Syndrome. OC43 = Human coronavirus OC43. SARS = Severe Acute Respiratory Syndrome.

Supplementary Procedure: Experimental design for in-tube fluorescent readout of *N* clinical samples with 20 μL reaction volumes.

A. Nuclease inactivation and viral particle lysis

1. Mix 2.28 * *N* μL TCEP-HCl (0.5 M) with 0.023 * *N* μL EDTA (0.5 M) at room temperature.
2. Add 8.8 μL of each sample [*e.g.*, nasopharyngeal swab in universal viral transport medium (UTM) or saliva] to a strip tube. Add 2 μL TCEP/EDTA mixture and 0.2 μL RNase inhibitor (40 U/uL) to each sample.
3. Incubate samples for 5 minutes at 40 ºC, followed by 5 minutes at 70 ºC (if UTM) or 5 minutes at 95 ºC (if saliva).

B. Viral RNA amplification and detection

For *N* samples with 20 μL reaction volumes and a 6.5% pipetting loss, the reaction factor *M* is *N*/5, rounded up to the nearest integer.

Reagent	Amount (μL)
RPA Pellets	1 * *M*
Reaction Buffer: 0.1 M HEPES pH 8.0, 300 mM KCl, 25% PEG-8000 (Optimized) (5X)	21.50 * *M*
Cas13a resuspended in SB (50 mM Tris pH 7.5, 600 mM NaCl, 5% glycerol, 2 mM DTT) (2.26 μM)	2.14 * *M*
6U FAM Reporter (2 μM)	3.19 * *M*
RNase Inhibitor (40 U/μL)	2.69 * *M*
rNTPs (25 mM each nucleotide)	8.60 * *M*
SuperScript IV Reverse Transcriptase (200 U/μL)	1.08 * *M*
RNase H (5 U/μL)	2.15 * *M*
T7 RNA Polymerase (50 U/μL)	2.15 * *M*
Nuclease-free H2O	50.60 * *M*
RPA Primers (5 μM each primer)	2.45 * *M*
Cas13a crRNA (2 μM)	0.46 * *M*
Magnesium Acetate (280 μM)	5.1 * *M*

1. Make a master mix of all components listed above, except the RPA pellets and the magnesium acetate. Keep the master mix on ice.

2. Resuspend the RPA pellets with 65 µL of the master mix each, returning the resuspended material to the master mix and mixing well.
3. Add magnesium acetate to the reaction mixture.
4. Aliquot 19 µL master mix into wells of a strip tube that is pre-chilled on ice. Add 1 µL of sample (*i.e.*, HUDSON product) or negative control (*e.g.*, nuclease-free water) to each aliquot, mixing thoroughly.
5. Incubate samples at 37 ºC for 30 to 90 minutes.
6. Download the companion smartphone application, which is available at https://github.com/sameeds/sherlock-reader-app. Follow instructions in the application to upload a picture of the results. The application will return binary outcomes (*i.e.*, whether each sample is positive or negative relative to the negative control).
