# Supplementary material for: Streamlined inactivation, amplification, and Cas13-based detection of SARS-CoV-2
Source: Nat Commun. 2020 Nov 20;11:5921. doi: 10.1038/s41467-020-19097-x (PMC7680145; doi:10.1038/s41467-020-19097-x)
Supplement: Supplementary file 4 — Reporting Summary [file 41467_2020_19097_MOESM4_ESM.pdf]

nature research

Corresponding author(s): Cameron Myhrvold

Last updated by author(s): 2020/08/18

Reporting Summary

Nature Research wishes to improve the reproducibility of the work that we publish. This form provides structure for consistency and transparency in reporting. For further information on Nature Research policies, see our Editorial Policies and the Editorial Policy Checklist.

Statistics

For all statistical analyses, confirm that the following items are present in the figure legend, table legend, main text, or Methods section.

n/a	Confirmed	
☐	☒	The exact sample size (*n*) for each experimental group/condition, given as a discrete number and unit of measurement
☒	☐	A statement on whether measurements were taken from distinct samples or whether the same sample was measured repeatedly
☐	☒	The statistical test(s) used AND whether they are one- or two-sided<br>*Only common tests should be described solely by name; describe more complex techniques in the Methods section.*
☒	☐	A description of all covariates tested
☒	☐	A description of any assumptions or corrections, such as tests of normality and adjustment for multiple comparisons
☐	☒	A full description of the statistical parameters including central tendency (e.g. means) or other basic estimates (e.g. regression coefficient) AND variation (e.g. standard deviation) or associated estimates of uncertainty (e.g. confidence intervals)
☐	☒	For null hypothesis testing, the test statistic (e.g. *F*, *t*, *r*) with confidence intervals, effect sizes, degrees of freedom and *P* value noted<br>*Give P values as exact values whenever suitable.*
☒	☐	For Bayesian analysis, information on the choice of priors and Markov chain Monte Carlo settings
☒	☐	For hierarchical and complex designs, identification of the appropriate level for tests and full reporting of outcomes
☒	☐	Estimates of effect sizes (e.g. Cohen's *d*, Pearson's *r*), indicating how they were calculated

Our web collection on statistics for biologists contains articles on many of the points above.

Software and code

Policy information about availability of computer code

Data collection	The code for the smartphone app is available on Github at https://github.com/broadinstitute/Handlens
Data analysis	Data panels were primarily generated via Prism 8 (GraphPad), except Figure 3E which was generated using Python (version 3.7.2), seaborn (version 0.10.1) and matplotlib (version 3.2.1).

For manuscripts utilizing custom algorithms or software that are central to the research but not yet described in published literature, software must be made available to editors and reviewers. We strongly encourage code deposition in a community repository (e.g. GitHub). See the Nature Research guidelines for submitting code & software for further information.

Data

Policy information about availability of data

All manuscripts must include a data availability statement. This statement should provide the following information, where applicable:

- Accession codes, unique identifiers, or web links for publicly available datasets
- A list of figures that have associated raw data
- A description of any restrictions on data availability

The authors declare that the data supporting the findings of this study are available in the Source Data File. The data, code, and detailed methods used in the design of primers and crRNAs are available at adapt.sabetilab.org. The code for the smartphone application analysis pipeline is available at https://github.com/broadinstitute/Handlens.

Field-specific reporting

Please select the one below that is the best fit for your research. If you are not sure, read the appropriate sections before making your selection.

☒ Life sciences ☐ Behavioural & social sciences ☐ Ecological, evolutionary & environmental sciences

For a reference copy of the document with all sections, see nature.com/documents/nr-reporting-summary-flat.pdf

Life sciences study design

All studies must disclose on these points even when the disclosure is negative.

Sample size	We determined that sample sizes of 2-6 technical replicates for synthetic target experiments was sufficient because the variation across replicates is small. No statistical methods were used to predetermine sample size. Experiments with patient samples usually contain a single biological and a single technical replicate per sample due to sample volume constraints and the inability to obtain biological replicates. When volume was available, patient sample experiments contained 3 technical replicates.
Data exclusions	Data were not excluded from any analyses.
Replication	Experiments were performed independently. Experiments with synthetic targets were replicated 2-4 times total, and all replication attempts were successful. Due to sample volume limitations, experiments with patient sample testing were only performed once.
Randomization	Randomization was not necessary as we do not run experiments in which we directly compare patient samples across two or more conditions (e.g., different testing strategies or regimes).
Blinding	Blinding was not necessary as we do not run experiments in which we directly compare patient samples across two or more conditions (e.g., different testing strategies or regimes).

Reporting for specific materials, systems and methods

We require information from authors about some types of materials, experimental systems and methods used in many studies. Here, indicate whether each material, system or method listed is relevant to your study. If you are not sure if a list item applies to your research, read the appropriate section before selecting a response.

Materials & experimental systems

n/a	Involved in the study	
☒	☐	Antibodies
☐	☒	Eukaryotic cell lines
☒	☐	Palaeontology and archaeology
☒	☐	Animals and other organisms
☒	☐	Human research participants
☒	☐	Clinical data
☒	☐	Dual use research of concern

Methods

n/a	Involved in the study	
☒	☐	ChIP-seq
☒	☐	Flow cytometry
☒	☐	MRI-based neuroimaging

Eukaryotic cell lines

Policy information about cell lines

Cell line source(s)	All cell lines were purchased from American Type Culture Collection (ATCC)
Authentication	No authentication was done beyond purchasing directly through ATCC
Mycoplasma contamination	All cell lines tested negative for mycoplasma.
Commonly misidentified lines (See ICLAC register)	None were used for this study.
