# Supplementary material for: Streamlined inactivation, amplification, and Cas13-based detection of SARS-CoV-2
Source: Nat Commun. 2020 Nov 20;11:5921. doi: 10.1038/s41467-020-19097-x (PMC7680145; doi:10.1038/s41467-020-19097-x)
Supplement: Supplementary file 5 — Description of Additional Supplementary Files [file 41467_2020_19097_MOESM5_ESM.pdf]

Title: Supplementary Data 1

Description: Detailed reaction conditions for optimization experiments.
